# A combined generalized Warblet transform and second order synchroextracting transform for analyzing nonstationary signals of rotating machinery

**DOI:** 10.1038/s41598-021-96343-2

**Published:** 2021-08-20

**Authors:** Kai Wei, Xuwen Jing, Bingqiang Li, Chao Kang, Zhenhuan Dou, Jinfeng Liu, Yu Chen, Hainan Zheng

**Affiliations:** 1 School of Mechanical Engineering, Jiangsu University of Science and Technology, Zhenjiang, 212013 China; 2 Shaanxi Diesel Heavy Industry. LTD, Xian, China

**Keywords:** Engineering, Mechanical engineering

## Abstract

In recent years, considerable attention has been paid in time–frequency analysis (TFA) methods, which is an effective technology in processing the vibration signal of rotating machinery. However, TFA techniques are not sufficient to handle signals having a strong non-stationary characteristic. To overcome this drawback, taking short-time Fourier transform as a link, a TFA methods that using the generalized Warblet transform (GWT) in combination with the second order synchroextracting transform (SSET) is proposed in this study. Firstly, based on the GWT and SSET theories, this paper proposes a method combining the two TFA methods to improve the TFA concentration, named GWT–SSET. Secondly, the method is verified numerically with single-component and multi-component signals, respectively. Quantized indicators, Rényi entropy and mean relative error (MRE) are used to analyze the concentration of TFA and accuracy of instantly frequency (IF) estimation, respectively. Finally, the proposed method is applied to analyze nonstationary signals in variable speed. The numerical and experimental results illustrate the effectiveness of the GWT–SSET method.

## Introduction

Rotating machinery is used in modern industry, civilian and military application, such as compressors, steam turbines, and aircraft engines. The tough working environment of rotating machinery leads to different types of faults in rotating machinery and increases the difficulty of fault diagnosis at the same time^1–4^. The vibration signal of rotating machinery is nonstationary, causing traditional Fast Fourier transform (FFT) cannot extract effective fault features. Time–frequency analysis (TFA) method is proposed to analyze non-stationary signals^5–7^. Unlike frequency transform, TFA methods can map a signal at time domain with one-dimension information into a signal at time–frequency domain with two-dimension information, which means it can portray the TF information of non-stationary signal to the TF distribution^8^. However, limited by the Heisenberg uncertainty principle^9^, the traditional TFA methods cannot obtain both high time resolution and high frequency resolution at the same time^10^. To overcome this shortage and improve the concentration of TFR, many effective TFA methods have been presented^11^.

The traditional TFA methods, like STFT, WVD, WT, representing Short-time Fourier transform, Wigner–Ville distribution and Wavelet Transform, respectively, are the basis of other various TFA methods, which belong to non-parametrized TFA methods. Based on these non-parametrized TFA methods, plenty of adaptive TFA methods have been studied. Mateo et al.^12^ improved time–frequency resolution by fixing the window size at frequency domain instead of at time domain. Yu et al.^13^ proposed an adaptive frequency band selection methods to filter noise signal. The frequency band range is calculated by the Particle Swarm Optimization (PSO), where the Harmonic Significance Index (HSI) is used to calculate the adaptation value of the PSO. Yuan et al.^14^ in order to eliminate the cross-infection term interference of the traditional WVD, sparse Bayesian learning (SBL) is used to decompose the signal, then the decomposed signal is analyzed using WVD. Those methods improved traditional TFA methods by making them adaptive and more effective to analyzing nonstationary signals. More recently, The parameterized TFA was proposed, compared to above adaptive TFA methods, the parameters of parameterized TFA can be changed according to the signal during analysis, and the initialized parameters are independent of the signal being analyzed.

Chirplet transform (CT), the classic parameterized TFA methods, is widely used due to the ability to perform effectively time–frequency analysis of liner chirp signal^15^. However, CT is not suitable for analyzing nonlinear signals. To overcome the shortage of CT, Peng et al.^16^ provide a new explanation of CT from the principle that CT is essentially a STFT on the signal by rotating and shifting the analyzed signal in the frequency domain with chirplet kernel. Hence, a polynomial chirplet kernel is used instead of the linear chirplet kernel to improve the effectiveness of CT for nonlinear signal analysis. However, the polynomial kernel exists Runge–Kutta phenomenon in dealing with complex signal, thus Yang et al.^17^ proposed a new chirplet kernel, named spline kernel. The experimental results show that Spline chirplet transform (SCT) can effectively avoid the Runge–Kutta phenomenon and obtain higher TFR concentration. The kernel functions in the above parametric time–frequency analysis methods are essentially function approximations of instantaneous frequencies, therefore these methods require high accuracy in the estimation of IF. Wang et al.^18^ explore the problem of IF estimation under noisy signal interference, and proposes to achieve signal decomposition by sparse filtering, thus improving IF estimation accuracy. Yang et al.^19^ used the Fourier series function named the Generalized Warblet transform (GWT) instead of sine function kernel used in conventional Warblet transform (CWT), and the experimental results show it is more effectively when processing the highly oscillating signal.

In addition to adaptive and parameterized TFA methods, post-processing techniques are employed to improve the concentration of TFR results as well. Post-processing techniques improve the concentration of TFR by reassigning the time–frequency coefficients of a raw TFR result into the proper instantly frequency trajectory positions^20^. Kodera et al.^21^ proposed reassignment method (RM), which offers an alternative approach to sharpen the TFR while keeping the temporal localization and are particularly well adapted to multicomponent signals. While, the main shortage of RM is the transform is invertible which means we cannot reconstruct the signal according to the calculated result^22^. Alternatively, Daubechies et al.^23^ proposed synchrosqueezing transform to keep the reversibility on the base of wavelet framework. Duong et al.^24^ to improve the concentration of TFR for a wide variety of amplitude signal, proposed the short-time Fourier combined with Synchrosqueezing Transform (SST) technology. Besides, Post-processing method, named synchroextracting transform (SET)^25^, is widely used in analyzing non-stationary signal recently. Differing from SST and RM, SET retains only the TF energy distribution on the IF trajectory by inner product the TFR results calculated by STFT with the Dirac δ function. SET is suitable for the analysis of multicomponent signals, but when the frequencies of the component signals are very close to each other, its TFA results appear diffusion phenomenon. Some other scholars proposed some effective TFA methods which are interesting. Sharma et al.^26^ using IEVDHM–HT to obtain the TFR of electroencephalogram (EEG) signal. Bhattacharyya et al.^27^ enhanced the empirical wavelet transform (EWT) by using Fourier–Bessel series expansion (FBSE), the Fourier–Bessel series expansion based empirical wavelet transform is an effective tool to analyzed signal with closed frequency components.

It can be seen from the literatures cited above that studies mainly focused on improve TFA methods to get a better TFR concentration. The GWT has a good accuracy in IF estimation due to the Fourier series function kernel. However, GWT is on the basis of the FM signal inner product the Fourier series kernel function, which means the concentration of TFR using GWT can be improved further by a post-processing technique (SSET).

In this paper, Generalized Warblet transform (GWT) coupled with Second Order Synchroextracting Transform (SSET) are employed to improve the concentration of TFA result. The contents of the study is organized as follows. A theory of GWT and SSET is demonstrated in “Theoretical background”. The proposed GWT–SSET is proposed in “GWT based SSET algorithm”. In “Numerical validation” and “Experimental validation”, the proposed method is validate by numerical and experiment respectively. Finally, the conclusions are drawn in “Conclusions” to summarize our work.

## Theoretical background

### Vibration signal and IF

A multicomponent amplitude-modulation and frequency-modulation (AM-FM) signal is described as^22^:

1 $$x(t) = \sum\limits_{k = 1}^{{\text{K}}} {x_{k} (t)} = \sum\limits_{k = 1}^{{\text{K}}} {A_{k} (t)e^{{{\text{j}}2\pi \phi_{k} (t)}} }$$

where *K* denotes the *x*(*t*) number of the mode and *A*_*k*_(*t*), *ϕ*_*k*_(*t*) represent the instantaneous amplitude and the instantaneous frequency (IF), respectively.

The target of a TFA method is to realize the ideal TFA. An ideal time–frequency representation (ITFR) of Eq. (1) can be described as^28^:

2 $$ITFR(t,\omega ) = \sum\limits_{k = 1}^{{\text{K}}} {A_{k} (t)\delta \left( {\omega - \phi_{k}^{^{\prime}} (t)} \right)}$$

The monocomponent signal is defined as Eq. (3) which is a liner chirp signal modulated by Gaussian^28^:

3 $$x(t) = A_{x} e^{{\frac{{\left( {t - t_{0} } \right)^{2} }}{{2s^{2} }}}} e^{{{\text{j}}2\pi \left( {{\text{a}} + {\text{b}}t + \frac{1}{2}{\text{c}}t^{2} } \right)}}$$

The derived value of *x*(*t*) with regards to *t* can be written as^28^:

4 $$\frac{{{\text{d}}x}}{{{\text{d}}t}}(t) = \left( {\frac{{\text{d}}}{{{\text{d}}t}}\left( {\ln \left( {A\left( t \right)} \right)} \right) + j\frac{{{\text{d}}\phi_{x} }}{{{\text{d}}t}}\left( t \right)} \right)x\left( t \right){\kern 1pt} = \left( {q_{x} t + p_{x} } \right)x(t)$$

With $$q_{x} = - \frac{1}{{s^{2} }} + jc$$ and $$p_{x} = \frac{{t_{0} }}{{s^{2} }} + jb$$.

According to Eq. (2), it can be known that the ideal TF ridge should coincide with the IF trajectory of the raw signal, and the TF amplitude of the TFR is the same as the instantaneous amplitude of the signal in the time domain.

### Generalized Warblet transform

Assuming that the Fourier series expansion of the period *T* and the frequency *ω*_1_ of function *f*(*x*) as^5,29^:

5 $$\begin{gathered} f(x) = \frac{{a_{0} }}{2} + \sum\limits_{i = 1}^{\infty } {A_{i} \left[ {\cos \phi_{i} \cos (n\omega_{1} x) + \sin \phi_{i} \sin (n\omega_{1} x)} \right]} \\ = \frac{{a_{0} }}{2} + \sum\limits_{i = 1}^{n} {a_{i} } \cos (i\omega_{1} x) + \sum\limits_{i = 1}^{n} {b_{i} } \sin (i\omega_{1} x) \\ \end{gathered}$$

where *a*_0_ = *A*_0_*, a*_*i*_ = *A*_*i*_*cos*(*ϕ*_*i*_)*, bi* = *A*_*i*_*sin*(*ϕ*_*i*_). Fourier series can not only describe periodic curves but also approximate any acyclic curve. A non-periodic function can be expanded into the Fourier series for the following conditions: the function is defined only in the support region and satisfies the Dirichlet sufficient conditions, that is, in a period, if there are intermittent points, the number of intermittent points is finite, while containing finite maximum and minimum values, and the function is absolutely productible^29^.

On the basis of this, The GWT of the analytic signal in Eq. (3) can be expressed as^19,30^:

6 $$GWT({\text{t}}_{0} ,\alpha ,\beta ,f,\omega ;\sigma ) = \int\limits_{ - \infty }^{ + \infty } {\overline{z} (t)w_{\delta } (t - t_{0} )e^{{ - {\text{j}}\omega t}} } {\text{d}}t$$

With

$$\left\{ \begin{gathered} \overline{{\text{z}}} (t) = {\text{x}}(t)\phi^{R} (t,\alpha ,\beta ,f)\phi^{S} (t,t_{0} ,\alpha ,\beta ,f) \hfill \\ \phi^{R} (t,\alpha ,\beta ,f) = \exp \left[ { - j\left( {\sum\limits_{i = 1}^{m} {\frac{{a_{i} }}{{f_{i} }}\cos 2\pi f_{i} t + } \sum\limits_{i = 1}^{m} {\frac{{\beta_{i} }}{{f_{i} }}\sin 2\pi f_{i} t} } \right)} \right] \hfill \\ \phi^{S} (t,t_{0} ,\alpha ,\beta ,f) = \exp \left[ {j2\pi \left( { - \sum\limits_{i = 1}^{m} {\alpha_{i} \sin 2\pi f_{i} t_{0} + } \sum\limits_{i = 1}^{m} {\beta_{i} \cos 2\pi f_{i} t_{0} } } \right)t} \right] \hfill \\ \end{gathered} \right.$$

In which *w*_*δ*_ is window function of STFT. *ϕ*^*R*^(*t,α,β,f*) is the frequency rotation operator which can rotate the analytic signal in frequency domain; *ϕ*^*S*^(*t,t*0*,α,β,f*) is the frequency which can shift the frequency component at time *t*_0_; *m* denotes the number of the functions. *{α*_1_*, α*_2_*… α*_*m*_*}* and *{β*_1_*, β*_2_* … β*_*m*_*}* are Fourier coefficients. {*f*_1_*, f*_2_* … f*_*m*_} is the corresponding frequencies. Since the kernel function is fitted to the IF, the more accurate the IF estimation, the higher TFR concentration. The TFR peak detection technique referred to^16^ is used to estimate IF trajectory.

### Second order synchroextracting

Second order synchroextracting (SSET), as a post-processing methods, can effectively enhance the energy distribution of TFR results by extracting the TF energy at the IF trajectory position. The STFT of *x*(*t*) with respect to the window *g*(*t*) is formulated as:

7 $$STFT_{x}^{g} \left( {t,\omega } \right) = \int\limits_{ - \infty }^{ + \infty } {x(\tau )} g^{*} \left( {\tau - t} \right)e^{{{\text{j}}\omega \left( {\tau - t} \right)}} {\text{d}}\tau$$

where the Gaussian window function with standard deviation *σ* is denoted by

8 $${\text{g}}\left( t \right) = \frac{1}{{\sqrt \sigma \sqrt[4]{\pi }}}e^{{\frac{{t^{2} }}{{2\sigma^{2} }}}}$$

The partial derivative of *STFTg’ x*(*t,ω*) with respective to *t* can be deduced as:

9 $$STFT_{{\text{x}}}^{g^{\prime}} (t,\omega ) = { - }q_{x} STFT_{x}^{tg} (t,\omega ) + \left( {q_{x} t + p_{x} - {\text{j}}\omega } \right)STFT_{x}^{g} (t,\omega )$$

The partial derivative of *STFTg’ x*(*t,ω*) with respective to *t* can be written as:

10 $$STFT_{x}^{g^{\prime\prime}} (t,\omega ) = - q_{x} STFT_{x}^{tg^{\prime}} (t,\omega ) + (q_{x} t + p_{x} - j\omega )STFT_{x}^{g^{\prime}} (t,\omega )$$

where *STFTg’’ x*, *STFTtg’ x* and *STFTg’ x* represent the STFTs using *g*^*’’*^(*t*), *tg*^*’*^(*t*) and *g*^*’*^(*t*) as a window function, respectively.

Combination the liner equations Eqs. (9) and (10), we can obtain:

11 $$\left\{ \begin{gathered} q_{x} t + p_{x} = \frac{{STFT_{x}^{g^{\prime}} STFT_{x}^{tg^{\prime}} - STFT_{x}^{g^{\prime}} STFT_{x}^{tg} }}{{STFT_{x}^{g} STFT_{x}^{tg^{\prime}} - STFT_{x}^{g^{\prime}} STFT_{x}^{tg} }} + {\text{j}}\varpi \hfill \\ q_{x} = \frac{{\left( {STFT_{x}^{g^{\prime}} } \right)^{2} - STFT_{x}^{g^{\prime\prime}} STFT_{x}^{g} }}{{STFT_{x}^{g} STFT_{x}^{tg^{\prime}} - STFT_{x}^{g^{\prime}} STFT_{x}^{tg} }} \hfill \\ \end{gathered} \right.$$

Thus the estimate of IF *ϕ*^*’*^(*t*) can be deduced as:

12 $$\hat{\phi }(t,\omega ) = \Im \left( {q_{x} t + p_{x} } \right)$$

Finally, the SSET can be defined as:

13 $$SSET = STFT_{x}^{g} \delta \left( {\omega - \phi^{\prime}(t)} \right)$$

## GWT based SSET algorithm

SSET, as a post-processing technique for GWT, is used to further enhance the TFR resolution. Taking the signal in Eq. (3), as an example, GWT–SSET can be expressed as:

14 $$GWTe({\text{t}}_{0} ,\alpha ,\beta ,f,\omega ;\sigma ) = \int\limits_{ - \infty }^{ + \infty } {x(t)\phi^{R} (t,\alpha ,\beta ,f)\phi^{S} (t,t_{0} ,\alpha ,\beta ,f)w_{\delta } (t - t_{0} )e^{{ - {\text{j}}\omega t}} }$$

Based on IF estimation technique according to Eqs. (11) and (12), the estimated IF of the signal can be written as

15 $$\begin{gathered} \hat{\phi }(t,\omega ) = \Im (q_{x} t + p_{x} ) \\ = \Im \left( {\frac{{GWTe_{x}^{g^{\prime}} GWTe_{x}^{tg^{\prime}} - GWTe_{x}^{g^{\prime}} GWTe_{x}^{tg} }}{{GWTe_{x}^{g} GWTe_{x}^{tg^{\prime}} - GWTe_{x}^{g^{\prime}} GWTe_{x}^{tg} }} + j\varpi } \right) \\ \end{gathered}$$

Finally, the TFR result of GWT–SSET can be formulated as:

16 $$GSSET_{S} \left( {t,\omega } \right) = GWT_{e} \cdot \delta \left( {\omega - \hat{\phi }\left( {t,\omega } \right)} \right)$$

**Figure 1:**
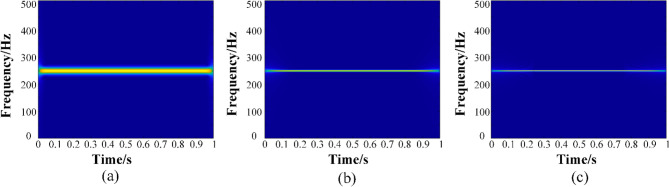
TFR results calculated by using (**a**) STFT, (**b**) GWT, (**c**) GWT–SSET.

A harmonic signal *s*(*t*) = *e*^2*iπ*(250*t*)^, where the IF is 250 Hz, is given below to test the performance of the proposed methods. With the frequency at the rate of 1024 Hz and the time duration is 1 s. The TFR results of the harmonic signal calculated by STFT, GWT and GWT–SSET are displayed in Fig. 1, respectively. With the same window length (1024) set, the frequency resolution of STFT is insufficient, GWT further improves the TFR resolution of the harmonic signal by constructing a suitable kernel function. As seen in Fig. 1c, the TFR concentration is further improved after post-processing SSET. The GWT (Fig. 1a) can effectively analyze monocomponent signals, but there is a problem of resolution degradation when analyzing multicomponent signals, which reduces the readability of TFR. Thus, an iterative technique is employed to realize the analysis of multicomponent signals according to Ref.^8^.

## Numerical validation

In this section, monocomponent signal and multicomponent signal are used to illustrate the TFR performance and IF estimation capability of the proposed methods. Other TFA methods are used to analyze the same signal, including STFT, SET, SSET, GWT, Fourier–Bessel series expansion based empirical wavelet transform and EVDHM–HT which are aimed to compare to the proposed methods.

### Monocomponent signal

The expression of the simulated monocomponent signal is:

17 $$s(t) = \sin \left( {200\pi t + 480\sin (t)} \right)$$

whose IF law is *IF*(*t*) = 100 + 240*cos*(*t*)*/π*. The sampling frequency at the rate of 500 Hz, and the time is 0–15 s.

**Figure 2:**
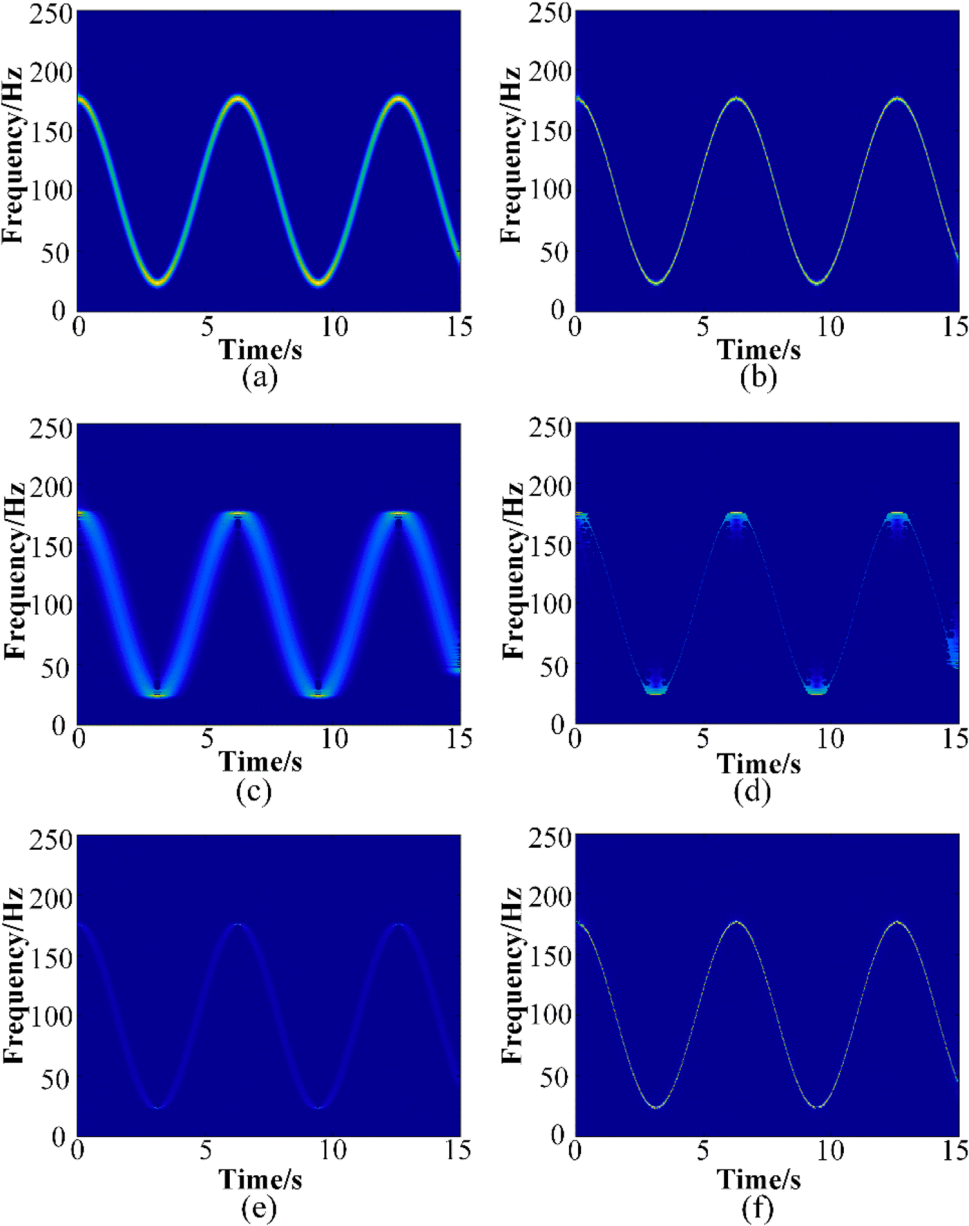
The TFRs obtained by (**a**) STFT, (**b**) GWT, (**c**) SET, (**d**) SSET, (**e**) SST, (**f**) GWT–SSET.

The TFRs of the test signal (17) calculated by GWT, STFT, SET, SSET, TSST and GWT–SSET are shown in Fig. 2 for comparison. The TF concentration of GWT is not as high as that of SSET, but there is frequency diffusion in TFR result calculated by SSET. GWT–SSET has the advantage of both methods of GWT and SSET, which means it has a high TF concentration without frequency diffusion.

We evaluated the proposed method by Rényi entropy as define in Eq. (18). Rényi entropy can be used to measure the energy concentration of the TFR. The formula for calculating Rényi entropy is as follows:

18 $$R_{\gamma } \left( {TF(t,\eta )} \right) = \frac{1}{1 - \gamma }\log_{2} \int\limits_{ - \infty }^{ + \infty } {\int\limits_{ - \infty }^{ + \infty } {TF^{\gamma } (t,\eta )} } {\text{d}}t{\text{d}}\eta$$

where *γ* = 3. The bigger the Rényi entropy, the worse the TFR concentration of TFA methods.

The Rényi entropy of TFR calculated by different TFA methods is listed in Table.1 for comparison. The smallest Rényi entropy is 5.0564 calculated by GWT–SSET. And the biggest Rényi entropy is 9.22 calculated by SET, which is higher 53.55% than the smallest. In addition, the Rényi entropy of GWT (6.73) higher 18.19% than the Rényi entropy of GWT–SSET, which indicates that GWT–SSET has a better TFR resolution. Therefore, it can be concluded that GWT–SSET has a better time–frequency resolution than the other TFA methods used in this paper.

**Table 1:** Rényi entropy of TFA results calculated by different methods for simulated signal.

Indictors	STFT	GWT	SET	SSET	SST	GWT–SSET
Rényi Entropy	7.7642	6.73	9.22	5.60	6.9113	5.0564

To test the IF estimation capability of the proposed method in a noisy environment, Gaussian white noise with different SNR is added to signal (17), and taking GWT and SSET as the comparison reference. Peak detection is used to extract IF, and the mean relative error is used to evaluate the IF estimation performance. The formula for calculating MRE is as follows:

19 $$MRE = \frac{1}{{N_{l} }}\left\| {\frac{{IF^{\prime} - IF}}{IF}} \right\|_{1}$$

where *N*_*l*_, *||·||*_1_, *IF* and *IF’* denotes the length of the *IF*, *l*_1_-norm, the original clean IF and the estimated IF, respectively.

**Figure 3:**
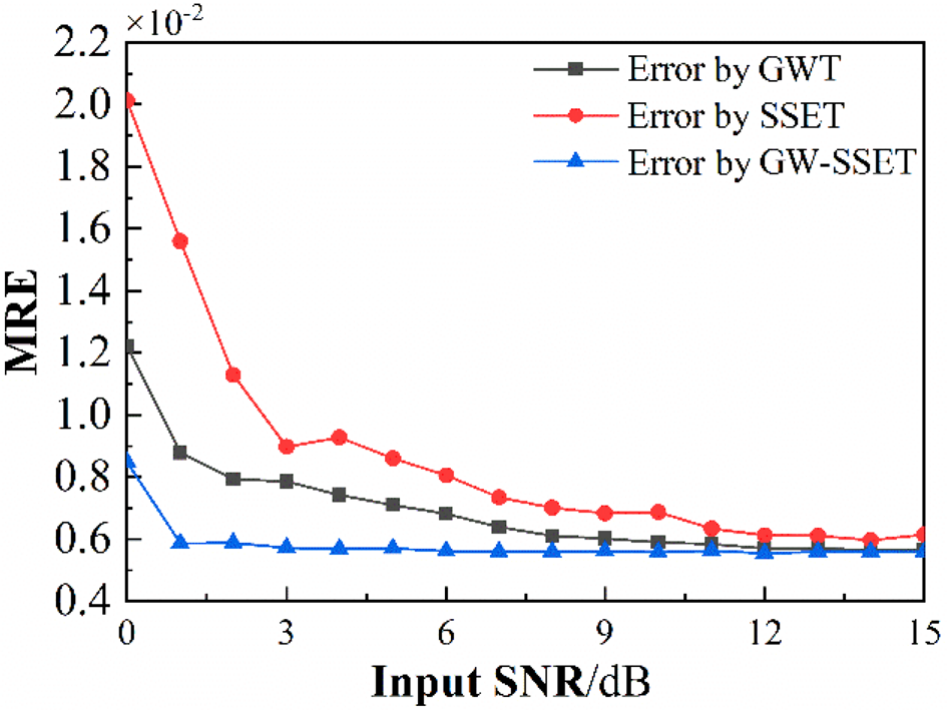
The errors of detected IF by GWT, SSET and GWT–SSET.

The MRE of estimated IF calculated by GWT, SSET and GWT–SSET are shown in Fig. 3. It can be seen that the MRE of GWT–SSET is always the smallest, especially in 0–3 dB, and the accuracy of the IF estimation by proposed method is better than that of GWT and SSET. This indicates that GWT–SSET is more accurate than the other two methods in IF estimation, which indicate that GWT–SSET is more robust to noise compared to other TFA methods.

**Figure 4:**
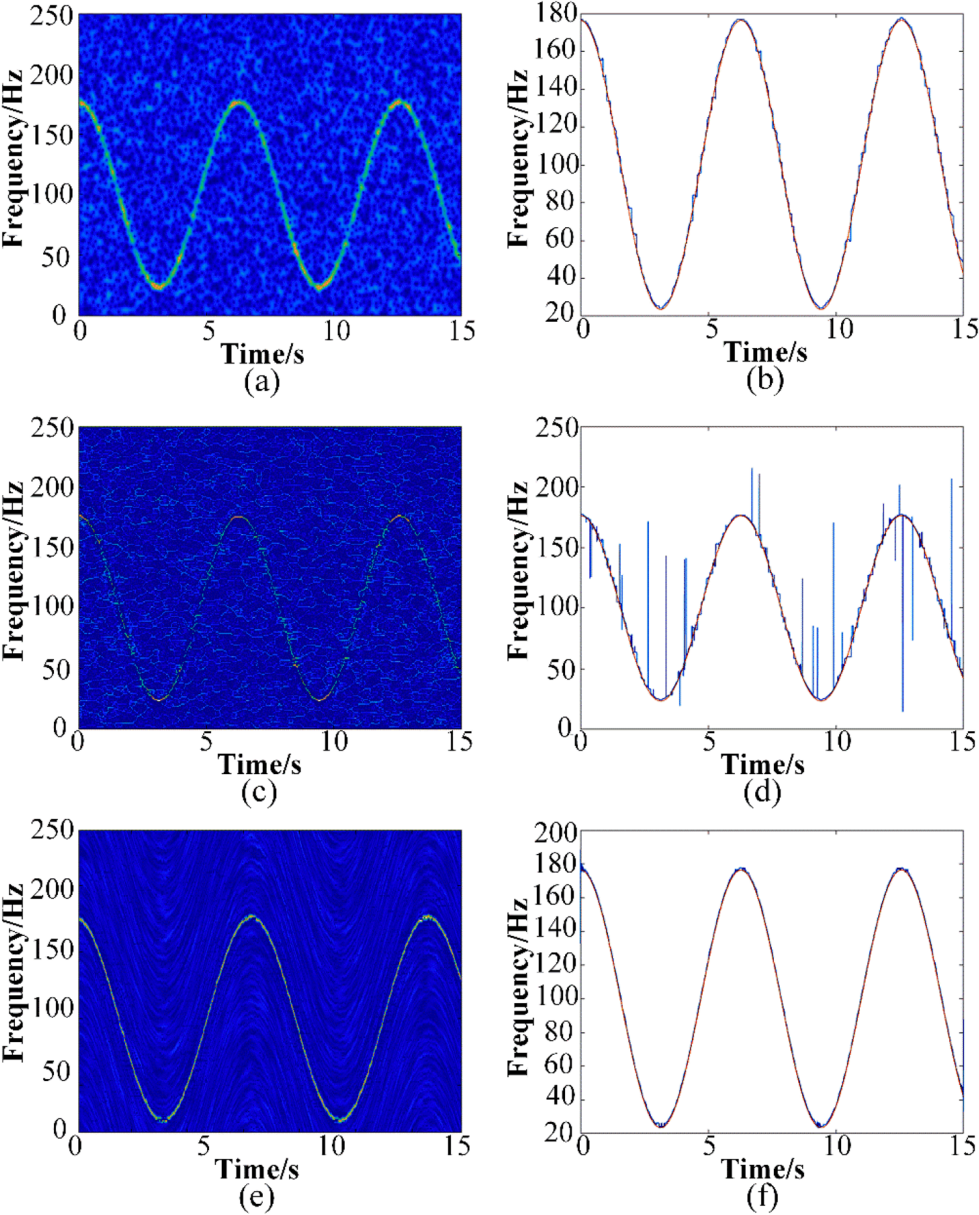
When SNR = 0 dB, (**a**) TFR result and (**b**) the IF detected by GWT, (**c**) TFR result and (**d**) the detected IF by SSET, (**e**) TFR result and (**f**) the detected IF by GWT–SSET, where the original IF (orange line), the estimated IF (blue line).

In addition, to illustrate the robustness of the proposed method to noise, the TFR and IF estimation (SNR = 0 dB) calculated by GWT, SSET and proposed methods are list in Fig. 4.It can be seen that the IF estimation calculated by SSET has a worse accuracy and exists severe frequency leakage. The IF estimated by GWT–SSET has a better accuracy and few frequency leakage compared to GWT and SSET.

### Multicomponent signal

To evaluate the performance of GWT–SSET in processing multicomponent vibration signal, a two-component nonstationary simulated signal is employed.

20 $$\begin{gathered} y_{1} (t) = s_{1} (t) + s_{2} (t) \hfill \\ s_{1} (t) = \cos (2\pi (20t + 4\cos (0.54t))) \hfill \\ s_{2} (t) = \cos (2\pi (f_{1} t + 0.4t^{2} )) \hfill \\ \end{gathered}$$

of which *IF*_1_(*t*) = 20*-*(54*sin*((27*t*)*/*50))*/*25 and *IF*_2_(*t*) = *f*_1_ + (4*t*)*/*5, *f*_1_ = 10 Hz, the sampling frequency at the rate of 200 Hz, and the time is 0–6 s.

**Figure 5:**
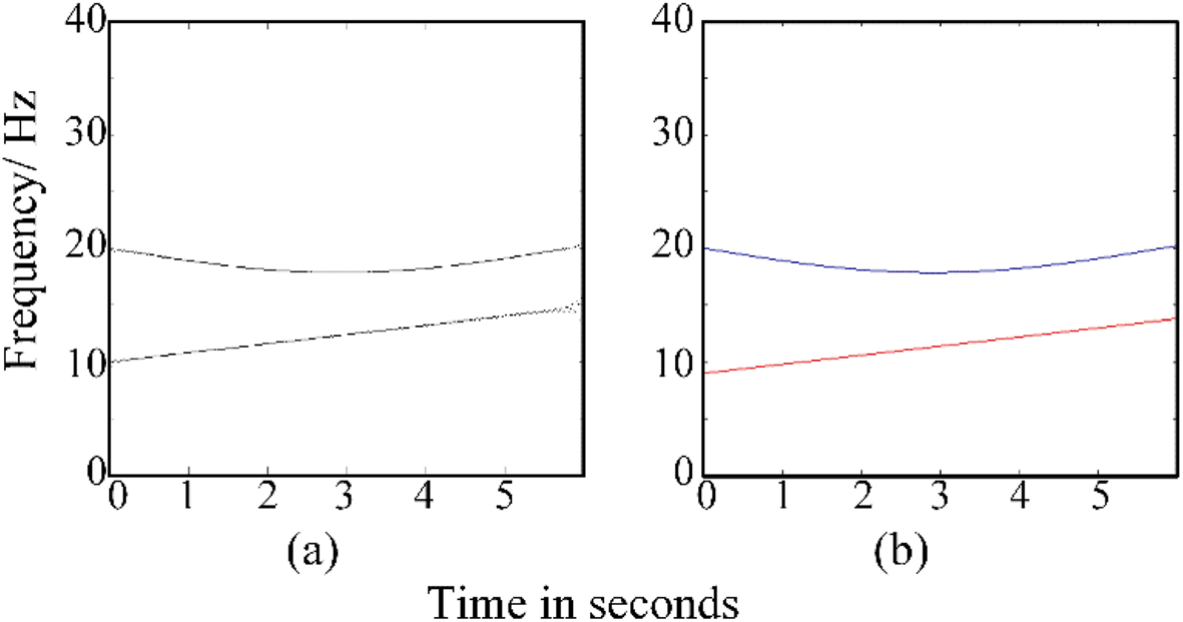
(**a**) The TFR result calculated by GWT–SSET, (**b**) The ideal IF.

The TFR result calculated by GWT–SSET and ideal IF of the non-stationary signal in Eq. (20) is shown in Fig. 5.

**Figure 6:**
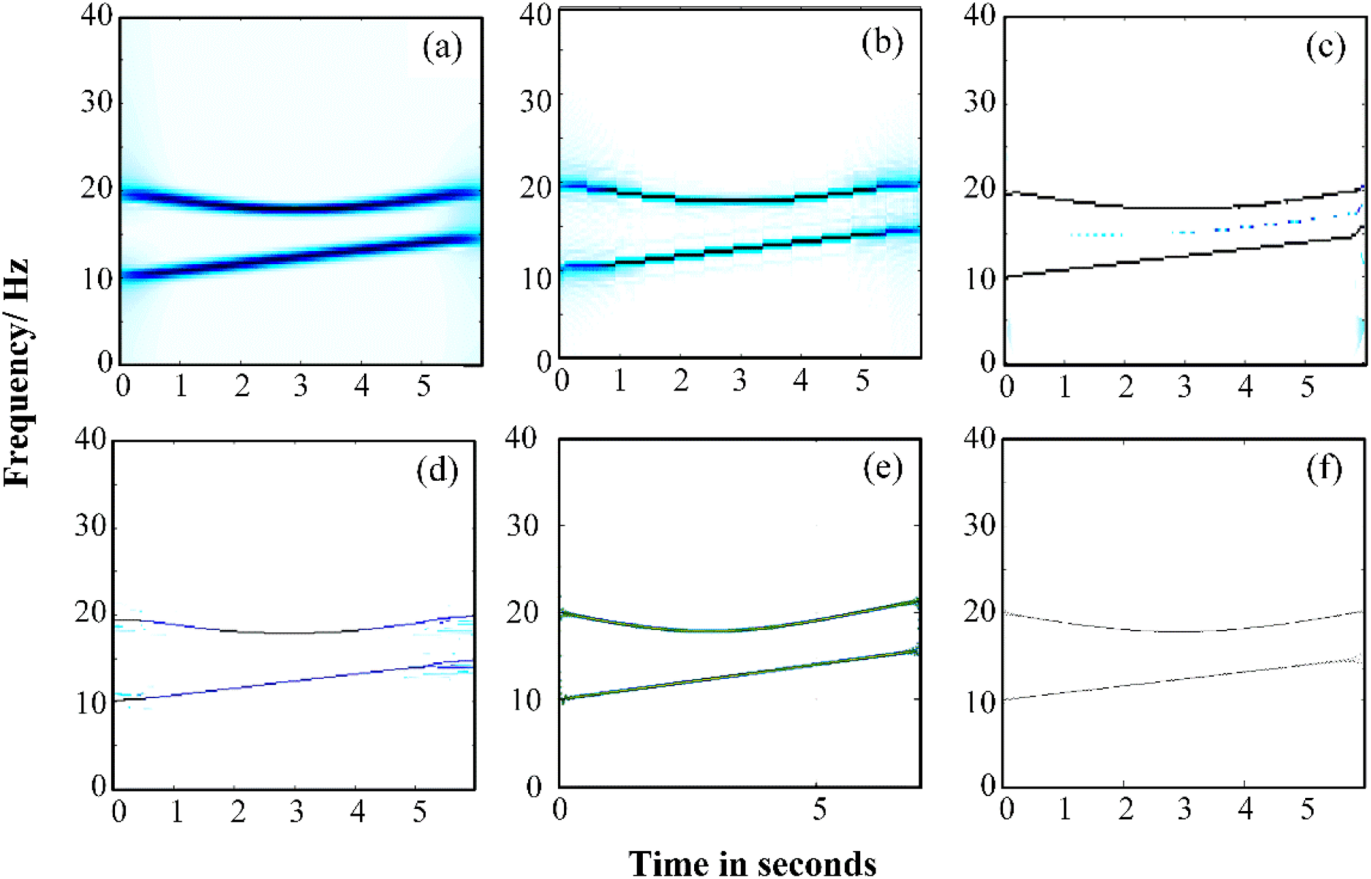
The TFR result calculated using (**a**) STFT, (**b**) GWT, (**c**) SET, (**d**) SSET (**e**) Fourier–Bessel series expansion based empirical wavelet transform (**f**) GWT–SSET.

For comparison, STFT, GWT, SET, SSET and Fourier–Bessel series expansion based empirical wavelet transform methods are employed to analyze the multicomponent signal. Figure 6 lists the TFR results calculated by various TFA methods. As can be seen from Fig. 6, the TFR resolution calculated by STFT is lowest (Fig. 6a), the resolution of TFR calculated by GWT and Fourier–Bessel series expansion based empirical wavelet transform (Fig. 6b,e) is relatively higher than the TFR calculated by STFT. However, they are not as high as that of SET and SSET, it can be seen that SET exists cross-term interface and SSET exists diffusion phenomenon (Fig. 6c,d). GWT–SSET (Fig. 6f) has a better TFR resolution without diffusion phenomenon and cross-term interface.

Furthermore, a Non-linear FM signal *y*_2_(*t*) is represented as^27^, to evaluated the robustness of GWT–SSET in noise by adding Gaussian white noise (SNR from 0 to 20 dB in step of 1).

21 $$\begin{gathered} y2({\text{n}}) = x_{1} (n - 100) + x_{2} (n - 100) \hfill \\ x_{1} (n) = A_{1} \cos ((a_{1} n^{3} + a_{2} n)(1/Fs)) \hfill \\ x_{2} (n) = A_{2} \cos ((a_{2} n\left| n \right| + a_{4} n)(1/Fs)) \hfill \\ \end{gathered}$$

where *A*_1_ = (497*/*4), *A*_2_ = (254*/*3), and [*a*1*, a*2*, a*3*, a*4] = [41*/*500*, *71*, *57*/*25*, -*4815*/*2]. The sample number is considered as 0 < n ≤ 199 and Fs = 1000 Hz.

**Figure 7:**
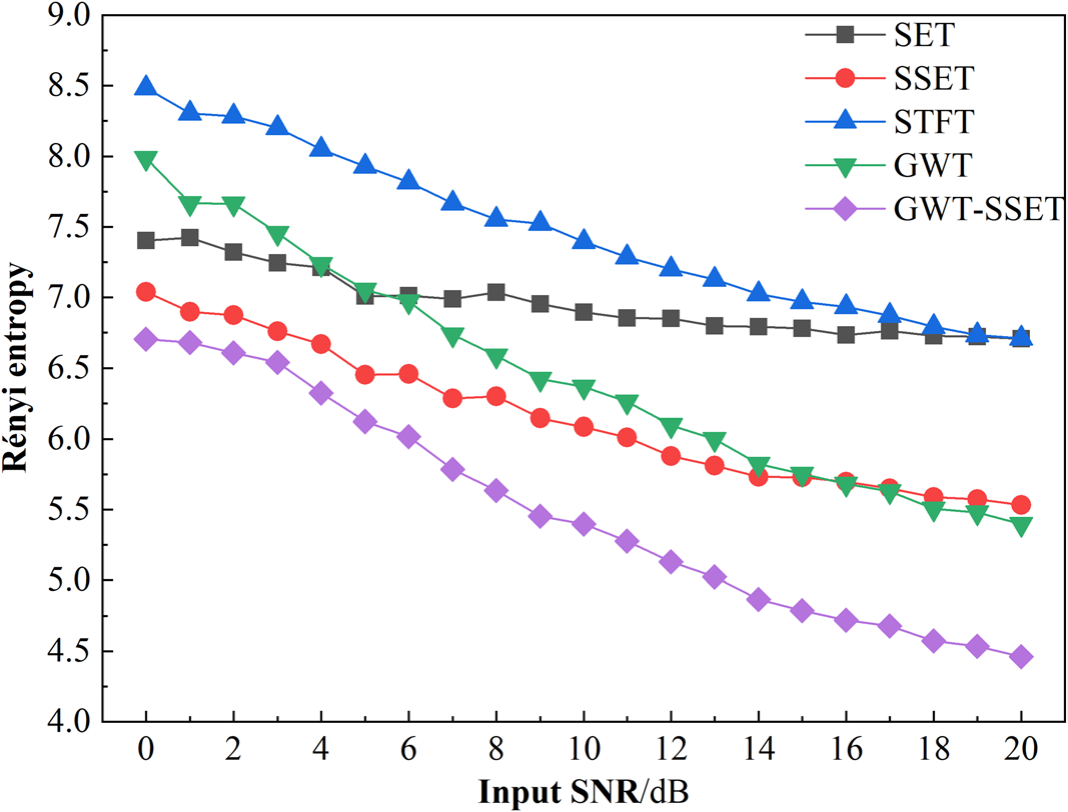
The Rényi entropy of TFR results.

The Rényi entropy calculated by different TFA methods under environmental noise is shown in Fig. 7. It can be seen that the Rényi entropy of GWT–SSET is always the smallest than other TFA methods used in this simulation. This indicates that the proposed methods has good robustness to vibration signals containing noise.

**Figure 8:**
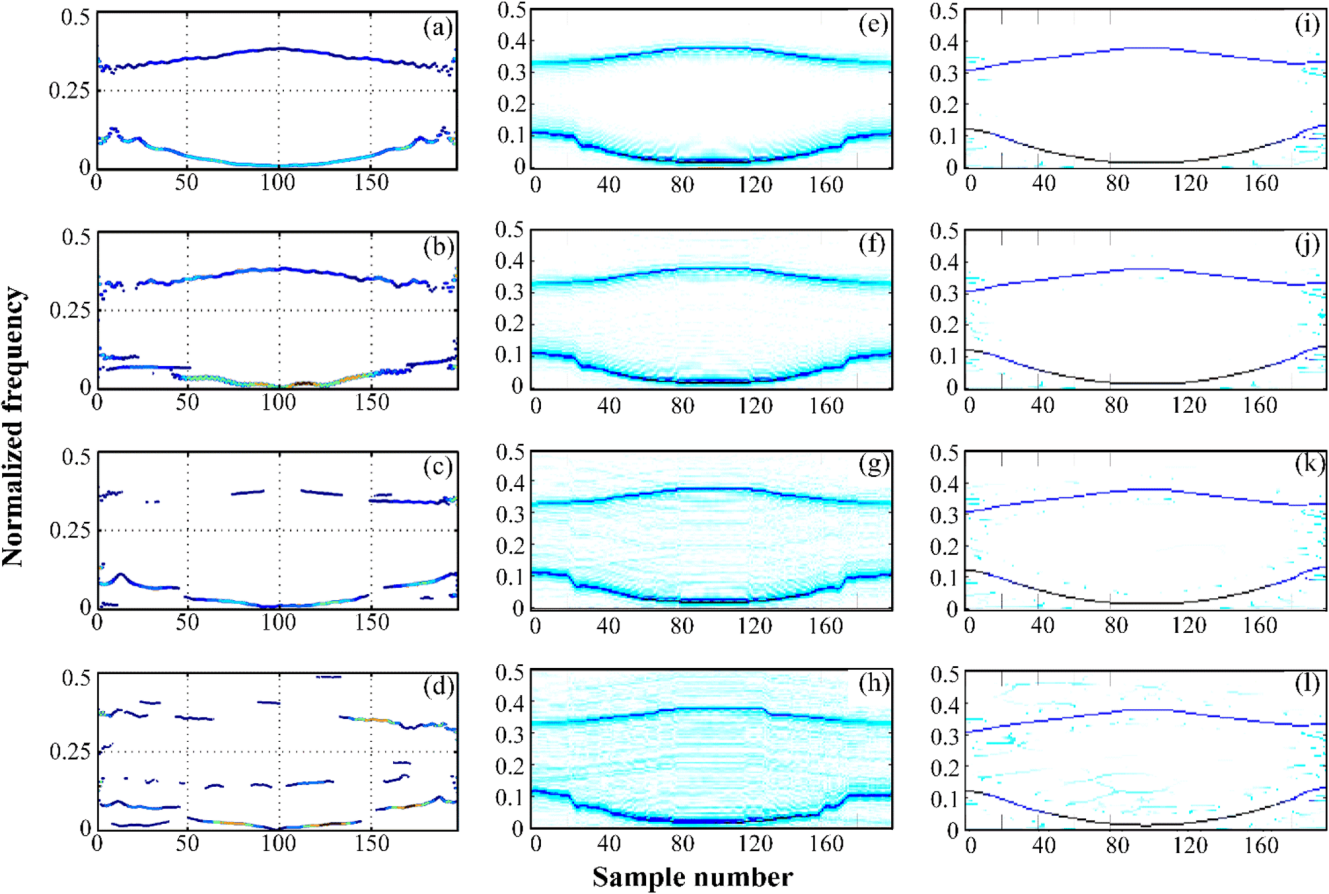
TFR of signal y4(n) using EVDHM–HT method, (**a**) Clean case, (**b**) SNR = 10 dB, (**c**) SNR = 5 dB, (**d**) SNR = 0 dB; using GWT method, (**e**) Clean case, (**f**) SNR = 10 dB, (**g**) SNR = 5 dB (**h**) SNR = 0 dB; using GWT–SSET, (**i**) Clean case, (**j**) SNR = 10 dB, (**k**) SNR = 5 Db, (**l**) SNR = 0 dB.

The TFR of signal *y*_2_(*t*) calculated by EVDHM–HT, GWT and GWT–SSET are depicted in Fig. 8. Figure 8a,e,i are the TFR of signal *y*_2_(*t*) under clean case, and the rest of figure are TFR of signal *y*_2_(*t*) under noisy case with noise levels 10, 5, and 0 dB SNRs. From Fig. 8, it can be seen that the resolution of TFR calculated by GWT is closed to that calculated by EVDHM–HT. However they are not as well as GWT–SSET under clean case. Under the noisy case, the EVDHM–HT cannot represent the components due to improper component merging criteria and use of multiple iterations^26^. It is evident that GWT–SSET not only has a better resolution of TFR but also has robustness in noisy.

## Experimental validation

**Figure 9:**
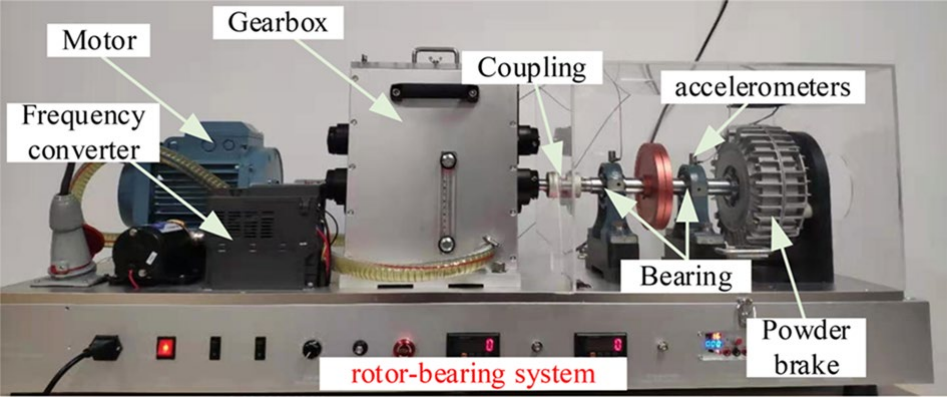
VALENIAN-PT500 The test rig.

In this paper, the VALENIAN-PT500 test rig is used to collect the bearing vibration data. The test rig is displayed in Fig. 9, consisting of a drive motor, a gearbox, a powder brake, a frequency converter and two bearing. Two acceleration sensors are mounted on the bearing housing to collect the vibration signal. The speed of test rig with a start-up about 1700–1900 rpm and a run-down about 1900–1100 rpm. Besides, there is no load torque added, and the sampling frequency at a rate of 1000 Hz. At the same time, the shaft rotating speed is monitor by an inductive sensor (tacho-meter).

**Figure 10:**
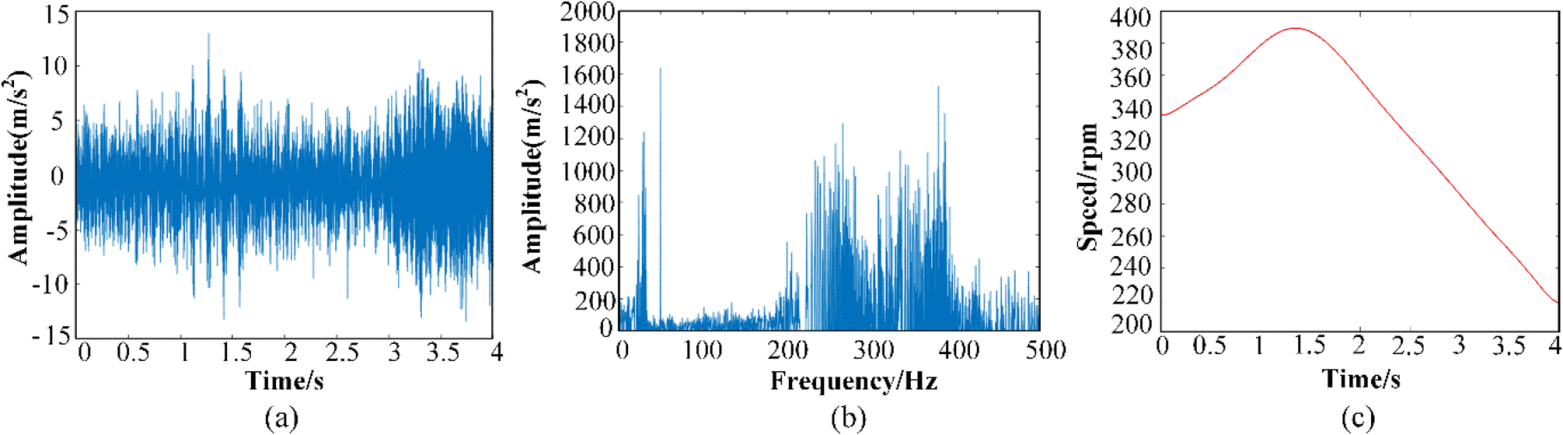
(**a**) The time domain spectrum (**b**) The frequency domain spectrum (**c**) motor speed.

**Figure 11:**
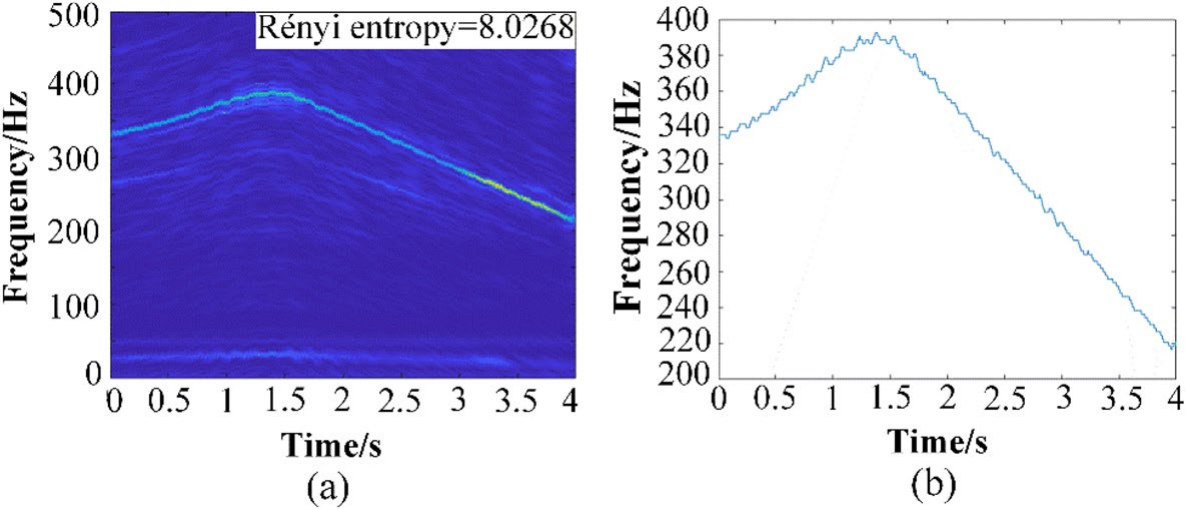
(**a**) TFR result calculated by GWT (**b**) estimation of instantaneous speed.

**Figure 12:**
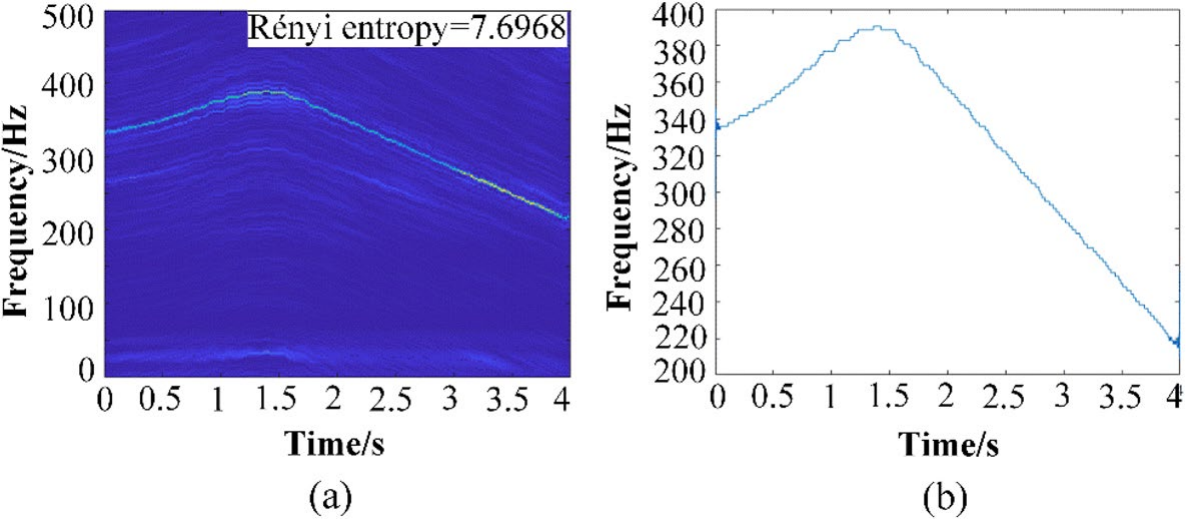
(**a**) TFR calculated by GWT–SSET (**b**) instantaneous speed estimation.

The collected signal and frequency spectrum of the original signal in 4 s are displayed in Fig. 10. Then the GWT and GWT–SSET are used to analyze the non-stationary signal. The corresponding TFR results are shown in Figs. 11a and 12a, respectively. The GWT–SSET provides a sharpen TFR, which can generate a better time–frequency location ability. The Rényi entropy calculated by the TFR of GWT–SSET is 7.6968. The Rényi entropy calculated by the TFR of GWT is 8.0268, which is bigger than GWT–SSET. It can be concluded that GWT–SSET has a better TFR resolution.

The IF ridge which extracted from TFR of GWT is shown in Fig. 11b, We can find that there are plenty of fluctuations in the result of instantaneous speed estimation, the ideal instantaneous speed should be smooth like Fig. 10c. The extracted IF ridge from TFR of GWT–SSET is shown in Fig. 12b, which is more accurate than GWT. Therefore, GWT–SSET has sufficient capability in analyzing AM-FM vibration signal characteristics.

## Conclusions

In this paper, a new TFA method is presented by combining GWT with SSET to improve the concentration of TFR result calculated by GWT. To evaluate effectiveness of the proposed methods, the numerical simulation is implemented. At the same time, Rényi entropy and MRE are employed as the indicator of TFR performance. Compared to other classical and advanced methods (STFT, PCT, GWT, SET and SSET), the simulated results show that the proposed method provides the TF distribution with better concentration. Next, we present applications of GWT–SSET in analyzing mechanical vibration data which acquired under varying speed condition. The experimental result indicating that GWT–SSET can get a better concentration of TFR results. Thus, it can be conclude that the proposed methods has potential for practical application, like frequency estimation and discrete signal analysis.
